# A retrospective study of the efficacy and safety of levofloxacin in children with severe infection

**DOI:** 10.3389/fped.2024.1381742

**Published:** 2024-04-05

**Authors:** Zhang Junqi, Cai Jie, Wang Jinglin, Lu Jinmiao, Lu Guoping, Wang Yi, Li Zhiping

**Affiliations:** ^1^ Department of Clinical Pharmacy, National Children’s Medical Center, Children’s Hospital of Fudan University, Shanghai, China; ^2^ Department of Pharmacy, Union Hospital, Tongji Medical College, Huazhong University of Science and Technology, Wuhan, China; ^3^ Pediatric Intensive Care Unit, National Children’s Medical Center, Children’s Hospital of Fudan University, Shanghai, China; ^4^ Department of Neurology, National Children’s Medical Center, Children’s Hospital of Fudan University, Shanghai, China

**Keywords:** levofloxacin, quinolone, children, infection, PICU

## Abstract

**Objectives:**

Levofloxacin is widely used because of its broad-spectrum antimicrobial activity and convenient dosing schedule. However, the relevance of its use in children remains to be investigated. The purpose of this study is to investigate the efficacy and safety of levofloxacin use in children with severe infections.

**Methods:**

We conducted a retrospective observational study of patients <18 years of age who received levofloxacin intravenously in the Pediatric Intensive Care Unit (PICU) of our hospital during the period between 2021 and 2022. Patient demographics, course characteristics, clinical effectiveness, and adverse event correlations were extracted through a retrospective tabular review.

**Results:**

We included 25 patients treated with 28 courses of levofloxacin. The mean age of these children treated with levofloxacin was 4.41 years. Conversion of pathogenic microbiological test results to negative after levofloxacin treatment was detected in 11 courses (39.29%). A decrease in inflammatory markers, white blood cell or C-reactive protein counts, was detected in 18 courses (64.29%). A total of 57 adverse events occurred during the treatment period, of which 21 were possibly related to levofloxacin and no adverse events were probably related to levofloxacin.

**Conclusion:**

The effectiveness of levofloxacin use in children with serious infections is promising, especially for the treatment of multidrug-resistant bacteria. Adverse events occurring during the initiation of levofloxacin therapy in children are reported to be relatively common, but in this study, only a small percentage of them were possibly related to levofloxacin, and none of them were highly possibly related to levofloxacin.

## 1 Introduction

Levofloxacin is a widely used antibiotic with significant antibacterial efficacy for various bacterial infections. Because of its broad-spectrum antimicrobial activity and convenient dosing schedule, levofloxacin is commonly recommended for the treatment of various infections, including respiratory tract infections, urinary tract infections, and gastrointestinal infections (1–4). It is also used for prophylaxis and treatment of febrile neutropenia in patients with hematological malignancies (5). However, its safety and efficacy in children, especially young children, is yet to be studied and often raises concerns (6, 7).

In China, levofloxacin's drug label is approved only for the treatment of inhalation anthrax and plague in patients aged 6 months and above, while drug labels in the EU and FDA have broader indications and can be used for the treatment of various pediatric diseases. With the increasing trend of pediatric drug-resistant infections, especially multidrug-resistant (MDR) and carbapenem-resistant *Enterobacter* (CRE) infections, multiple authoritative academic organizations have issued guidelines or reached a consensus in recent years to provide evidence for off-label use of drugs by clinicians (8–10). In China, pharmaceutical experts have developed an expert consensus on the use of fluoroquinolones in children to regulate their use in pediatrics (11). The Management of Community-Acquired Pneumonia in Infants and Children Older Than 3 Months of Age: Clinical Practice Guidelines by the Pediatric Infectious Diseases Society and the Infectious Diseases Society of America (2011), recommends that for adolescents with skeletal maturity, levofloxacin (500 mg once daily) or moxifloxacin (400 mg once daily) can be used as an alternative treatment for *Mycoplasma pneumoniae* pneumonia (8). The 2023 IDSA Antibiotic Resistance in Gram-Negative Infections treatment guidance document also proposes that fluoroquinolones may be considered as a treatment option for drug-resistant infections (12).

However, fluoroquinolones can cause joint lesion in juvenile animals, and this effect is related to both dosage and duration of treatment (13). There are species differences between animals and humans, and further investigation is needed to explore the safety of these drugs in children (14, 15). This retrospective cohort study aims to analyze the efficacy and safety of levofloxacin in children with severe infections and provide some empirical references for its use in such children, as well as discuss the safety of quinolones in pediatric severe infections.

## 2 Methods

### 2.1 Study design

We performed a retrospective study of patients who received levofloxacin injection in the Pediatric Intensive Care Unit (PICU) at the Children’s Hospital of Fudan University. All patients who were treated with levofloxacin had been off the drug for at least 1 year.

### 2.2 Inclusion and exclusion criteria

We included all patients <18 years of age who were administered at least one dose of intravenous levofloxacin in the PICU between 2020 and 2021.

### 2.3 Data collection, study definitions, and assessment of adverse events

All information on patient demographics, chronic conditions, and details of levofloxacin use was obtained from the medical records of the hospital. Adverse events (AEs) were identified by prespecified criteria (detailed later).

We used one course of levofloxacin as a unit of analysis, which is defined as ≥1 consecutive days of levofloxacin treatment. On the basis of the half-life of levofloxacin, >97% of the drug is eliminated within 2 days of a dose; we therefore analyzed periods of therapy separated by a gap of >2 days as separate courses. We define medications taken at the same time as the first dose of levofloxacin as “scheduled medications”.

We considered the laboratory values obtained ≤1 week before the first dose of levofloxacin as the baseline values for each course. The AEs we recorded included any discomfort that occurred during levofloxacin treatment, and ≤2 days after discontinuation. The AEs recorded by the treatment provider in the medical documentation at the time of the occurrence of the AE was the primary attribution of the AE. If no such attribution was documented, then laboratory abnormalities were identified by using predefined reference values from the Harriet Lane Handbook. The AEs identified during the levofloxacin courses that began before the initiation of the drug were attributed to another drug or underlying condition, as appropriate. Correlations between AEs and levofloxacin were resolved by using the Naranjo Adverse Drug Reaction Probability Scale (NADRPS).

### 2.4 Statistical analysis

Excel was used to establish a database, and SPSS 25.0 statistical software was used for statistical analysis. Normally distributed measurement data were expressed as mean ± standard deviation, while measurement data with a skewed distribution pattern were expressed as median (IQR, 25th–75th percentile).

## 3 Results

### 3.1 Demographic data

A total of 28 levofloxacin courses were initiated in 25 patients during the study period (Table 1). Infection is the main factor involved in the use of levofloxacin. In this study, congenital anomalies were found to be the most common chronic conditions among patients [12 (42.9%)], followed by respiratory system anomalies [10 (35.71%)].

**Table 1:** Patient characteristics.

Patient characteristics	Value
Age, mean (range)	4.41 years (3 months–14 years)
Sex (male), *n* (%)	17 (60.71)
Drug combination (*n*), average (range)	1.7 (0–6)
Chronic condition institution, *n* (%)	
Neoplasm	5 (17.86)
Endocrine, nutritional, and metabolic disease and immunity disorder	10 (35.71)
Disease of blood and blood-forming organ	5 (17.86)
Disease of the nervous system and sense organ	6 (21.43)
Disease of the circulatory system	6 (21.43)
Disease of the respiratory system	10 (35.71)
Disease of the digestive system	10 (35.71)
Disease of the musculoskeletal system	3 (10.71)
Congenital anomalies	12 (42.86)

### 3.2 Levofloxacin-course characteristics

The characteristics of the 28 levofloxacin courses were studied (Tables 2, 3). The average duration of therapy was 12.11 days. The average dose prescribed (14.19 mg/kg/day) was consistent with the dosing recommended by the World Health Organization. Of the 28 courses, levofloxacin was chosen for use when patients did not respond to conventional treatment. Three of the courses involved the use of empiric therapy [3 (10.71%)], and most of the remaining courses, except one, involved the treatment of gram-negative infections (Table 3).

**Table 2:** Prescription characteristic.

Prescription characteristic	Value
Duration of therapy (days), mean (median, range)	12.11 (10,2–28)
Courses per patient (*n*), median (range)	1 (1–2)
Dose (mg/kg/day), mean (range)	
6 months to <5 years	16.05 (8–20)
≥5 years	10.89 (8–16)
Premature discontinuation for any adverse event, *n* (%)	1 (3.57)

**Table 3:** Drug therapy characteristics.

Drug therapy characteristics	Value
Strain classification	
Gram-positive bacteria, *n* (%)	6 (21.43)
Gram-negative bacteria, *n* (%)	20 (71.43)
Microbiological test results turned negative, *n* (%)	11 (39.29)
White blood cell count decreased, *n* (%)	14 (50.00)
C-reactive protein decreased, *n* (%)	13 (46.43)

### 3.3 Efficacy of levofloxacin

We also studied the efficacy of levofloxacin (Table 3). Of the 28 courses, 23 (82.14%) involved the treatment of those infected with gram-negative bacteria and 6 (21.43%) involved the treatment of those infected with positive bacteria. After levofloxacin treatment, white blood cell count (WBC) decreased in 14 (50.00%) courses, and C-reactive protein (CRP) decreased in 13 (46.43%). Conversion of pathogenic microbiological test results to negative after levofloxacin treatment was detected in 11 courses (39.29%). Eighteen (64.29%) of these courses showed a decrease of WBC or CRP. These results indicate that most patients treated with levofloxacin experienced a decrease in inflammatory markers.

### 3.4 AEs and monitoring

Among all 28 courses, a total of 57 AEs occurred; 21 (28.07%) AEs involving 10 courses were possibly associated with levofloxacin (Table 4) and none were probably related to levofloxacin. The most common AEs possibly associated with levofloxacin were direct bilirubin elevation [4 (14.29%)] and alanine aminotransferase elevation [4 (14.29%)], followed by an increased aspartate aminotransferase level [3 (10.71%)]. Elevated levels of these markers suggest that they may be related to the liver function of the patients. Our study did not find a clear difference between younger and older children in terms of the incidence of adverse events that may be associated with levofloxacin.

**Table 4:** Adverse events noted during the initiation of levofloxacin therapy.

Adverse event	Total occurrences of adverse events, *n* (%)	Possible adverse events associated with levofloxacin, *n* (%) of 28 courses
Increased alanine aminotransferase level	5 (17.86)	4 (14.29)
Increased aspartate aminotransferase level	9 (32.14)	3 (10.71)
Rash	2 (7.14)	2 (7.14)
Increased total bilirubin level	8 (28.57)	2 (7.14)
Increased direct bilirubin level	11 (39.3)	4 (14.29)
Increased alkaline phosphatase level	1 (3.57)	0 (0.00)
Abdominal pain	2 (7.14)	0 (0.00)
Diarrhea	2 (7.14)	0 (0.00)
Hyperglycemia	1 (3.57)	0 (0.00)
Increased creatinine level	5 (17.86)	0 (0.00)
Increased creatine kinase isoenzymes level	2 (7.14)	0 (0.00)
Peripheral neuropathy	4 (14.29)	1 (3.57)
Death	3 (10.71)	0 (0.00)

## 4 Discussion

In 2019, approximately 4.95 million people died from drug-resistant bacterial disease, of which 1.27 million deaths were directly attributable to drug-resistant pathogens (16, 17). With the increase in drug-resistant bacterial disease, the Chinese 2023 National Guidelines for Antimicrobial Therapy state that when no other low-toxicity and high-efficiency antimicrobial drugs are available, especially for severely affected children, quinolones may be chosen on balance of probability.

We retrospectively studied 28 courses of levofloxacin in 25 children with severe infections and found that levofloxacin was generally highly effective and safe. The three main bacteria detected in our study were *Stenotrophomonas maltophilia* (*n* = 9), *Acinetobacter baumannii* (*n* = 6), and *Burkholderia cepacia* (*n* = 5). In our study, the etiological conversion rate of *Stenotrophomonas maltophilia* treated with levofloxacin was 44.44% (4/9), which was lower than 81.6% in the study by Nys et al. (18). This difference may be attributed to the fact that, first, all patients in our study were from the PICU and their clinical condition was more severe than those of others; second, our study was retrospective in nature, and some patients were not retested through an etiological examination after treatment. Although the etiological negative conversion rates of *Acinetobacter baumannii* and *Burkholderia cepacia* reached 50% and 80%, respectively, levofloxacin was not recommended as a therapeutic agent in the clinical guidelines and medication methods, and therefore, we suggest that levofloxacin is not the mainstay of treatment for eradication of these two bacteria.

In this study, we use the NADRPS to evaluate the AEs, which was first proposed by Naranjo et al. in 1981 (19). It has been recognized by several publications for its advantage of achieving a balance between ease of use and scientific validity (20, 21). The NADRPS establishes 10 evaluation indicators, scores each indicator individually, and assesses the causality level based on the final score. The evaluation results include categories such as “doubtful,” “possible,” “probable,” and “certain.” However, due to the limitations of the clinical application, some evaluation processes may allow for the selection of the unknown option, which may result in a low number of ADRs evaluated as “certain.” Overall, the evaluation results in this study confirmed that there was no significant difference between the NADRPS and the World Health Organization-Uppsala Monitoring Center (WHO-UMC) scale (22). Adverse events occurring during the initiation of levofloxacin therapy in children are reported to be relatively common, but in this study, only a small percentage of them were possibly related to levofloxacin, and none were probably related to levofloxacin. We found that direct bilirubin elevation was the most common AE attributed to levofloxacin, followed by alanine aminotransferase elevation, both of which are recognized effects of fluoroquinolones. In a study of >1,700 children, Hampel et al. showed that the incidence rate of total adverse events in children taking ciprofloxacin was 18.9% (drug-related and non-drug-related), and the most common adverse events were gastrointestinal reactions (e.g., diarrhea, nausea, and vomiting), followed by headache and abdominal pain, as well as arthralgia in 1.5% of patients (23). The probability of total adverse events and adverse events with higher incidence rates differed to some extent from that of our study because, first, levofloxacin and ciprofloxacin are slightly different, even though both are quinolones; second, the probability difference can be attributed to the fact that our study subjects were children with severe infections with a higher probability of adverse events occurring because of their more complex conditions and a higher liver burden with more coadministered medications.

Current studies on the use of levofloxacin in children have focused primarily on the prophylaxis of infections and the evaluation of safety, and these studies have shown good prophylactic efficacy and no increase in toxic effects (24–26). Our study shows the efficacy of levofloxacin in children with severe infections and the absence of adverse events that were probably related to it, complementing the gap in research on the use of levofloxacin in this population.

We acknowledge certain limitations in our study. Because our study included patients from the PICU, their clinical condition was usually complex; therefore, the study results might not be generalizable to healthy children and adverse effects could not be easily judged. We used retrospective studies, and all adverse events were derived from physician records or laboratory results obtained when these studies were performed, making it difficult to detect new adverse effects. Our study did not follow up with these children to determine whether they experienced long-term skeletal muscle toxicity. However, a large prospective clinical study indicates that levofloxacin causes skeletal muscle damage that is comparable to that of controls and appears to be reversible (27). In addition, due to the limitations of the retrospective study design, pathogenic microbiological data could not be collected from all patients and an appropriate control group was not included. This might affect the validity of the efficacy evaluation. Therefore, prospective studies are necessary to determine the safety and efficacy of levofloxacin in children with severe infections.

## Data Availability

The raw data supporting the conclusions of this article will be made available by the authors without undue reservation.

## References

[1] Bush LM Kaye D. Catheter-associated urinary tract infection IDSA guidelines: why the levofloxacin? Clin Infect Dis. (2010) 51(4):479–80; author reply 480–1. 10.1086/655159 20635873

[2] Emmi V. Guidelines for treatment of pneumonia in intensive care units. Infez Med. (2005):7–17. PMID: 16801748

[3] Kranz J Schmidt S Lebert C Schneidewind L Vahlensieck W Sester U Epidemiology, diagnostics, therapy, prevention and management of uncomplicated bacterial outpatient acquired urinary tract infections in adult patients: update 2017 of the interdisciplinary AWMF S3 guideline. Urologe. (2017) 56(6):746–58. 10.1007/s00120-017-0389-1 28455578

[4] Zagari RM Frazzoni L Marasco G Fuccio L Bazzoli F. Treatment of *Helicobacter pylori* infection: a clinical practice update. Minerva Med. (2021) 112(2):281–7. 10.23736/S0026-4806.20.06810-X 32700868

[5] Lehrnbecher T Fisher BT Phillips B Alexander S Ammann RA Beauchemin M Guideline for antibacterial prophylaxis administration in pediatric cancer and hematopoietic stem cell transplantation. Clin Infect Dis. (2020) 71(1):226–36. 10.1093/cid/ciz1082 31676904 PMC7312235

[6] Jantarabenjakul W Suntarattiwong P Wacharachaisurapol N Supradish Na Ayudhya P Phaisal W Tawan M Pharmacokinetics and safety of WHO-recommended dosage and higher dosage of levofloxacin for tuberculosis treatment in children: a pilot study. Int J Infect Dis. (2022) 122:603–8. 10.1016/j.ijid.2022.07.029 35842213

[7] Noel GJ Bradley JS Kauffman RE Duffy CM Gerbino PG Arguedas A Comparative safety profile of levofloxacin in 2523 children with a focus on four specific musculoskeletal disorders. Pediatr Infect Dis J. (2007) 26(10):879–91. 10.1097/INF.0b013e3180cbd382 17901792

[8] Bradley JS Byington CL Shah SS Alverson B Carter ER Harrison C The management of community-acquired pneumonia in infants and children older than 3 months of age: clinical practice guidelines by the Pediatric Infectious Diseases Society and the Infectious Diseases Society of America. Clin Infect Dis. (2011) 53(7):e25–76. 10.1093/cid/cir531 21880587 PMC7107838

[9] Chow AW Benninger MS Brook I Brozek JL Goldstein EJ Hicks LA IDSA clinical practice guideline for acute bacterial rhinosinusitis in children and adults. Clin Infect Dis. (2012) 54(8):e72–112. 10.1093/cid/cis370 22438350

[10] Guarino A Ashkenazi S Gendrel D Lo Vecchio A Shamir R Szajewska H. European Society for Pediatric Gastroenterology, Hepatology, and Nutrition/European Society for Pediatric Infectious Diseases evidence-based guidelines for the management of acute gastroenteritis in children in Europe: update 2014. J Pediatr Gastr Nutr. (2014) 59(1):132–52. 10.1097/MPG.0000000000000375 24739189

[11] Wu JY Sun SM. Expert consensus on the use of fluoroquinolones in children. Pharmacy Today. (2018) 28(1):10.

[12] Tamma PD Aitken SL Bonomo RA Mathers AJ van Duin D Clancy CJ. Infectious Diseases Society of America 2023 guidance on the treatment of antimicrobial resistant gram-negative infections. Clin Infect Dis. (2023) 74(12):2089–114. 10.1093/cid/ciab1013 37463564

[13] Yabe K Satoh H Ishii Y Jindo T Sugawara T Furuhama K Early pathophysiologic feature of arthropathy in juvenile dogs induced by ofloxacin, a quinolone antimicrobial agent. Vet Pathol. (2004) 41(6):673–81. 10.1354/vp.41-6-673 15557076

[14] Hampel B Hullmann R Schmidt H. Ciprofloxacin in pediatrics: worldwide clinical experience based on compassionate use—safety report. Pediatr Infect Dis J. (1997) 16(1):127–9; discussion 160–2. 10.1097/00006454-199701000-00036 9002122

[15] Bacci C Galli L de Martino M Chiappini E. Fluoroquinolones in children: update of the literature. J Chemother. (2015) 27(5):257–65. 10.1179/1973947815Y.0000000054 26099190

[16] Laxminarayan R. The overlooked pandemic of antimicrobial resistance. Lancet. (2022) 399(10325):606–7. 10.1016/S0140-6736(22)00087-3 35065701 PMC8769677

[17] Antimicrobial Resistance Collaborators. Global burden of bacterial antimicrobial resistance in 2019: a systematic analysis. Lancet. (2022) 399(10325):629–55. 10.1016/S0140-6736(21)02724-0 35065702 PMC8841637

[18] Ny C Cherabuddi K Venugopalan V Klinker KP. Clinical and microbiologic outcomes in patients with monomicrobial *Stenotrophomonas maltophilia* infections. Antimicrob Agents Chemother. (2019) 63(11):e00788–19. 10.1128/aac.00788-19 31427300 PMC6811402

[19] Naranjo CA Busto U Sellers EM Sandor P Ruiz I Roberts EA A method for estimating the probability of adverse drug reactions. Clin Pharmacol Ther. (1981) 30(2):239–45. 10.1038/clpt.1981.154 7249508

[20] Agbabiaka TB Savović J Ernst E. Methods for causality assessment of adverse drug reactions: a systematic review. Drug Saf. (2008) 31(1):21–37. 10.2165/00002018-200831010-00003 18095744

[21] Arimone Y Bégaud B Miremont-Salamé G Fourrier-Réglat A Molimard M Moore N A new method for assessing drug causation provided agreement with experts’ judgment. J Clin Epidemiol. (2006) 59(3):308–14. 10.1016/j.jclinepi.2005.08.012 16488362

[22] Pandit S Soni D Krishnamurthy B Belhekar MN. Comparison of WHO-UMC and Naranjo scales for causality assessment of reported adverse drug reactions. J Patient Saf. (2024). 10.1097/PTS.0000000000001213 38345209

[23] Grady R. Safety profile of quinolone antibiotics in the pediatric population. Pediatr Infect Dis J. (2003) 22(12):1128–32. 10.1097/01.inf.0000101994.25947.12 14688586

[24] Davis A Stevens AM Brackett J Marquez L Foster CE Sauer HE Levofloxacin prophylaxis for pediatric leukemia patients: longitudinal follow-up for impact on health care-associated infections. Pediatr Blood Cancer. (2022) 69(7):e29525. 10.1002/pbc.29525 35029328

[25] Karol SE Sun Y Tang L Pui CH Ferrolino J Allison KJ Fluoroquinolone prophylaxis does not increase risk of neuropathy in children with acute lymphoblastic leukemia. Cancer Med. (2020) 9:6550–5. 10.1002/cam4.3249 32710497 PMC7520302

[26] Wolf J Tang L Flynn PM Pui CH Gaur AH Sun Y Levofloxacin prophylaxis during induction therapy for pediatric acute lymphoblastic leukemia. Clin Infect Dis. (2017) 65:1790–8. 10.1093/cid/cix644 29020310 PMC5850441

[27] Bradley JS Kauffman RE Balis DA Duffy CM Gerbino PG Maldonado SD Assessment of musculoskeletal toxicity 5 years after therapy with levofloxacin. Pediatrics. (2014) 134:e146–53. 10.1542/peds.2013-3636 24918220

